# Challenges faced by marginalized communities such as transgenders in Pakistan

**DOI:** 10.11604/pamj.2018.30.96.12818

**Published:** 2018-06-05

**Authors:** Hassan Bin Usman Shah, Farah Rashid, Iffat Atif, Muhammad Zafar Hydrie, Muhammad Waleed Bin Fawad, Hafiz Zeeshan Muzaffar, Abdul Rehman, Sohail Anjum, Muhammad Bin Mehroz, Ali Haider, Ahmed Hassan, Hassaan Shukar

**Affiliations:** 1 Department of Community Medicine, Yusra Medical & Dental college, Islamabad (YMDC), Pakistan; 2 Joint program of family and community medicine, Directorate of Public Health, Ministry of Health Jeddah Region, Jeddah, Kingdom of Saudi Arabia

**Keywords:** Commercial sex work, gender based discrimination, hijras, institutional discrimination, physical violence, risk behaviors, social exclusion, suicide, transgender

## Abstract

**Introduction:**

Stigmatization, social exclusion and consequent banishment from the society makes transgender's life even tougher; isolating, pushing and forcing them into inappropriate conducts/habits like selling sex. This study investigates the association of social exclusion/victimization with high-risk behaviors among transgender community of Rawalpindi and Islamabad (Pakistan).

**Methods:**

Through a cross-sectional study design, a sample of 189 transgender community living in twin cities of Rawalpindi and Islamabad was selected using snowball sampling technique. A validated close ended questionnaire was used to estimate the high-risk behaviors. Multivariate logistic regression was used to explore the competing outcomes associated with suicidal risk, selling sex and substance abuse.

**Results:**

Majority study participants 77.8% experienced physical attacks with institutional discrimination even higher (91.5%). Commercial sex work and drug abuse was reported in 39.2% and 37.6% respectively. The prevalence of suicide ideation was high (38.6%) however, suicide attempted rate was less (18.5%). In the multivariate logistic regression, compared to those with no risk, being physically attacked increased the odds of both attempting (OR=2.18) and contemplating suicide and selling sex (OR=4.10). Nevertheless, the relative impact of institutional victimization on suicidal behavior was higher among those who were targeted on the basis of their gender identity or expression (AOR = 6.20, CI = 1.58-24.29, p=0.009).

**Conclusion:**

The transgender community is socially excluded by the Pakistani society where they experience high levels of physical abuse and face discriminatory behavior in daily life. Such attitudes make them vulnerable for risky behaviors; forcing them to become commercial sex workers, begging, drugs use and even suicidal ideation.

## Introduction

Trans sexuality and transgender communities have gained little visibility in our society as we are taught to believe in strict binaries of male and female genders. We tend to consider them taboo, distancing ourselves and humiliating them without trying to understand their problems [1]. In Pakistan, the most commonly used word for the transgender is 'Hijra'; an umbrella term covering various forms of gender deviances. It covers anyone who bends the common societal constructions of gender including cross-dressers, homo and bisexuals, true hermaphrodites, eunuch, transsexuals, transvestites, genderqueer youth, drag queens and transgender [1]. Injustice, poverty, illiteracy, social and cultural boycott, lack of opportunities and non-dominant social identities are some reasons for social exclusion for this marginalized group. This cornered minority have the extra stress of coping with their sexual orientation in our society [2]. Studies show that social exclusion is pushing the transgender community into inappropriate conducts/habits [2,3]. Most of the members of this jeopardized minority are forced to indulge in risky and dangerous ways of earning their livelihood, including sex work, dancing, begging and substance abuse etc [1,3]. Literature shows that vulnerable groups who are engaged in one problematic behavior are more likely to engage in others also [2]. We need to rethink about these forgotten people and listen to their plight before they reach a point of no return (i.e. more suicidal ideation and tendency) [4]. Furthermore, once their sexual orientation is disclosed they may face harassment, violence and lifetime of repeated victimization not only from the society, in schools from class fellows, and even by the other members of their home. Thus, the already stigmatized and cornered group is subjected to unending and systematic oppression [5]. Studies conducted with transgender population demonstrated violent physical victimization rates ranging from 43% to 60% [6,7]. Such factors force them to run away from their home, increase drop outs from schools, violating laws and indulging in activities which can harm not only their but others health too; common being prostitution, and I/V drugs use [6,7].

Moreover, suicidal ideation and attempts has been identified as an additional life threatening issue associated with victimization [8]. A four-fold increase in suicidal ideation is seen in transgender associated with physical victimization [9]. Once transgender leave their house or are forced to do so; they start living together in the traditional commune arrangement of five or more disciples (commonly famous as “Chelas”) supervised by a “Guru (teacher).” Each house has its own history, rules and identified by its Guru. Once transgender reaches there, training in singing, dancing and other activities are given enabling them to earn a livelihood. The Guru is responsible to manage the funds of his house and meet chelas needs; so, the chelas are expected to turn their earnings over to the guru. [1,10]. Most of the times guru is unable to make both ends meet and chelas start indulging in commercial sex for financial support [10] Moreover, with increasing modernity the income generating role from dancing and blessing births, has declined, forcing them into begging and sex trade [1,10]. Rarely someone would opt sex work as a profession by choice because of the stigma, discrimination, religious prohibition and the risks attached to it [1,11]. In most of the cases they are forced to sell sex to earn a living, making themselves vulnerable and a source of spreading of sexually transmitted diseases [1,12]. It is important to understand the social context and identify the causes of these risk behaviors which are pushing transgender to inappropriate conducts/habits. Early recognition of the factors leading to increase in substance abuse, forcing transgender into commercial sex and suicidal tendency can bring change in their lives. This will help in designing an effective intervention that would aim to improve the behaviors, protect the people based on their gender identity and expression [11,12]. This study summarizes the diverse factors and gives an insight of how and what this group themselves experience and paints a picture of how our social exclusion is pushing them to cross the line.

## Methods

A cross-sectional study was carried out in different locations of Rawalpindi and Islamabad. The total duration of the study was 6 months, from 1^st^ June to 1^st^ Dec 2016. The data was collected from 189 transgender people selected through snow ball sampling technique. Sample size for the transgender people included in this survey was calculated using Epitools sample size calculator. Calculated sample size was 139 by keeping expected transgender population at 10% (taken from other regional studies) [10,13] with 0.05% desired precision and 95% confidence level. Researchers approached 207 transgender people but n= 18 (8.6%) refused to participate, so the response rate of 91.4% was noticed. Before the interview multiple meetings with the Gurus were held to develop a rapport and to get permission from the decision maker of the house (i.e. Guru). The interviews were conducted privately mostly at their place of residence since they are usually suspicious on strangers and this helped in breaking the ice and gave the participants a chance to express themselves completely. Information was obtained through direct interview from the transgender, using standardized close ended questionnaires whose components were incorporated from internationally standardized instruments [5,10,13,14]. This validated close ended structured questionnaire; translated in local language with slight modification was used. It was translated from English into Urdu, by two bilingual professional translators who understood content. The translated instrument was then back- translated into English by two other bilingual translators and compared to its original version. This procedure ensured clarity and comprehensibility of items. Any discrepancies in comparison were discussed and a few minor adjustments were applied after pilot-testing. Data on demographics, identification as being transgender, source of income, housing, substance abuse, sexual history, risk behaviors, family and society support, receiving psychotherapy, physical victimization and gender based discrimination along with suicidal tendency was gathered from the study participants. Along with these, questions related to community's behavior, treatment given at the health care facilities and law enforcing agency's behavior were also included.

Discrimination based victimization was assessed on responses to two question [14]; including ever been physically/verbally attacked (like jeered, grabbed, punched, stabbed, hit by a rock etc). An affirmative response led to question identifying/asking gender identity being primary reason for these attacks. We derived two measures from responses of above mentioned questions. A confirmation that gender identity being main cause for these attacks was coded as discrimination based victimization (yes = 1; no = 0); and non-discriminatory victimization, if reported physical attack was not due to transgender status (coded as yes = 1; no = 0). Similarly, any institutional discrimination experienced by the respondent was also assessed. These include being fired/denied from the job, lost and denied housing, discrimination at the hospital, school and denied enrollment in insurance schemes etc. (coded as yes = 1; no = 0). For this study, we defined suicide risk in three ways; suicidal ideation only (thought about suicide but never attempted), suicidal ideation total (who actively considered suicide including those who attempted) and suicidal behavior (who had attempted suicide but not think about it anymore). If transgender have neither contemplated nor attempted suicide ever, they were classified as “No risk for suicidal behavior” which served as our reference group. (All were coded as yes = 1; no = 0) [14]. The interviews with the participants were done during the day when the participants were not working and available to participate in the study. Moreover, they were also provided with information regarding spread of infection and modes of prevention. Eligibility criteria included individuals 18 years or older and willing to provide informed consent for this study. Ethical approval was taken from ethical review board of Yusra Medical & Dental College (ERC# YMDC/01/17/ERB/109). Data analysis was done using SPSS 22. Frequencies and percentages were calculated for the categorical variables. Bivariate logistic regression analysis was conducted to determine whether discriminatory victimization, non-discriminatory victimization and institutional discrimination, forced sex and community/family support (in addition to several co- variates) are related to suicide risk, sexual behavior and substance abuse. Multivariate regression analysis was also conducted while controlling for sociodemographic and psychosocial variables.

## Results

The mean age of our study population was 29 ± 7.88 years. Around 94.2% (n=178) were unemployed. Majority (65.6%) were having monthly income of less than 10000; much less than the government's new laid-down minimum pay scale of Rs. 14,000/month (announced in 2016 budget). More than 90% were living in rented house in groups headed by Guru. Recreational drug use was reported by n=71 (37.6%) of the transgender; marijuana and I/V drugs being commonest. Only n=46 (24.3%) and n=52 (27.5%) had knowledge about the diseases associated with needle sharing and unprotected sex respectively. Seeking health care from government hospitals was negligible (9.5%), they prefer going to private (mostly NGO based/traditional healers) setups (70.4%). Mean age at 1st sex (mostly forced) was 15 ± 2.41 years. Main source of income was beggary, dance and alms (69.3%) however, 39.2 % also reported selling sex for their livelihood. Other demographic profile is given in Table 1. Discriminatory behavior towards transgender community not only by the family but also at community level; whether in hospital, schools, professional organizations etc. forces them into the corner, developing negative attitude. Details are shown in Table 2. Table 3 shows bivariate and multivariate regression analysis for the prediction of risk factors. In bivariate analysis, discriminatory victimization was associated with an increased risk of substance abuse (OR = 3.81, CI=1.07-13.52, p =0.038) and selling sex (OR=4.10, CI =1.15-14.54, p=0.029). Compared to no risk, institutional discrimination was associated with increased risk of suicidal ideation (only/total) and attempts (Table 3). Selling sex and begging are also significantly associated with institutional discrimination. Similarly, forced sex at young age was significantly associated with future habit of selling sex (OR=6.47, CI= 3.09-13.53, p <0.001). Lack of support from the family and community was associated with a higher likelihood of suicidal ideation total (OR=4.24, CI=1.84-9.78, p=0.001) (Table 3). The multivariate regression analysis indicated that those who experienced institutional discrimination and faced physical discrimination continued to be at a higher risk for suicidal ideation, substance abuse and selling sex (Table 3).

**Table 1:** Demographic profile and characteristics of transgender individuals (n=1890029`

Variables	Number (n)	Percentage (%)
Lack of awareness about their rights	117	61.9
No formal education or less than 5^th^ grade	139	73.5
No health education or psychotherapy session	156	82.5
Alcohol use	45	23.8
Living with Gurus/group	176	93.1
Satisfied with Guru’s attitude	161	85.2
Temporary residence (Not in stable housing)	163	86.2
Institutional discrimination	173	91.5
Suicidal thought (no attempt)	73	38.6
Suicidal attempt	35	18.5
Selling sex	74	39.2
Substance abuse (other than alcohol)	71	37.6
Begging	143	75.7

**Table 2:** Reasons/factors responsible for negative attitude in transgender community(n=189)

Variable	Number (n)	Percentage (%)
Criticism/jeer from people	147	77.8
Expectation of prostitution from you	135	71.4
Society’s/community’s behavior enforce you to isolate	151	79.9
Discrimination by doctors	72	38.1
Unsatisfactory treatment in the hospitals	121	64.0
Offensive behavior of law enforcement agencies	143	75.7
Disrespectful behavior by the customer	139	63.5
Physically abused in last 1 year	147	77.8
Not easy to find rented place	153	81.0
Gurus force you to sell sex	60	31.7
Forced sex	117	61.9

**Table 3:** Bivariate and multivariate regression predicting risk behaviors among transgender community

Variable	Unadjusted OR (95% CI)	*p* value	Adjusted OR (95% CI)	*p* value
**Discriminatory victimization**				
Suicidal ideation only	1.533 (.561-4.186)	0.405	0.815 (.178-3.719)	0.815
Suicidal ideation (total)	2.121 (.824-5.461)	0.119	1.110 (.251-4.905)	0.890
Suicide attempt	2.184 (.483-9.881)	0.311	1.765 (.194-16.064)	0.614
Selling Sex	4.105 (1.159-14.543)	0.029	4.214 (.784-22.642)	0.094
Substance Abuse	3.815 (1.076-13.522)	0.038	8.946 (1.023-78.230)	0.048
Begging	3.694 (1.428-9.560)	0.007	1.512 (.365-6.263)	0.568
**Non-discriminatory victimization**				
Suicidal ideation only	0.579 (.148-2.255)	0.431	0.510 (.066-3.956)	0.519
Suicidal ideation (total)	0.416 (.118-1.473)	0.174	0.444 (.062-3.182)	0.419
Suicide attempt	0.424 (.052-3.422)	0.420	0.719 (.035-14.869)	0.831
Selling Sex	0.144 (0.18-1.148)	0.067	0.542 (.036-8.209)	0.659
Substance Abuse	0.351 (.074-1.673)	0.189	2.727 (.188-39.519)	0.462
Begging	0.242 (.070-8.33)	0.024	0.374 (.052-2.699)	0.329
**Institutional discrimination**				
Suicidal ideation only	4.873 (1.074-22.106)	0.040	4.704 (.930-23.788)	0.061
Suicidal ideation (total)	6.531 (1.795-23.769)	0.004	6.202 (1.583-24.297)	0.009
Suicide attempt	3.669 (.468-28.749)	0.216	3.821 (.461-31.694)	0.214
Selling Sex	4.990 (1.100-22.637)	0.037	4.160 (.774-22.344)	0.097
Substance Abuse	2.806 (.771-10.214)	0.117	2.122 (.534-8.435)	0.285
Begging	6.343 (2.161-18.614)	0.001	5.700 (1.711-18.990)	0.005
**Forced sex**				
Suicidal ideation only	1.441 (.781-2.659)	0.242	1.350 (.695-2.622)	0.375
Suicidal ideation (total)	1.174 (.650-2.121)	0.595	1.015 (.523-1.971)	0.964
Suicide attempt	0.784 (.372-1.651)	0.521	0.701 (.315-1.560)	0.384
Selling Sex	6.470 (3.094-13.530)	0.000	8.554 (3.777-19.376)	0.000
Substance Abuse	2.009 (1.068-3.780)	0.031	2.000 (1.021-3.914)	0.043
Begging	0.730 (.362-1.472)	0.379	0.609 (.269-1.377)	0.233
**Family and community supportive**				
Suicidal ideation only	2.590 (1.057-6.346)	0.037	2.856 (1.117-7.298)	0.028
Suicidal ideation (total)	4.245 (1.841-9.789)	0.001	4.992 (2.078-11.994)	0.000
Suicide attempt	3.992 (.907-17.571)	0.067	4.325 (.958-19.522)	0.057
Selling Sex	2.659 (1.086-6.512)	0.032	3.324 (1.208-9.146)	0.020
Substance Abuse	1.398 (.620-3.153)	0.420	1.139 (.478-2.715)	0.769
Begging	1.044 (.433-2.516)	0.924	1.225 (.452-3.323)	0.690

The final sample size was 189

No suicidal risk, not selling sex, not using substance abuse and not begging are the referent category

## Discussion

The purpose of this article was to examine the association between the experiences of discrimination (physical, institutional and societal) and indulging of these sexual minorities into risky and dangerous ways of earning their livelihood and the occurrence of suicidal and no suicidal self- harm. Our findings provide evidence that transgender community living in Pakistan face extreme form of social exclusion mainly because of negative attitude of people towards them as shown by Ahmed et al study [1]. Discriminatory victimization, institutional discrimination, physical/verbal abuse, forced sex at the tender age and lack of support from the family and community all have the potential for sizeable effects on the risky behaviors; including high rates of suicidal ideation/attempts in transgender, selling sex and drug abuse [15]. Owing to these, our study population were involuntarily compelled to cross the line where 76% reported begging and 39% selling sex; multivariate analysis identifying social exclusion the main culprit. Similar sinful and inappropriate conducts/habits as a reaction of social exclusion were also reported in other studies conducted in Pakistan, Bangladesh & India [1,12,13,15,16]. Around 80% of our study sample reported being attacked physically/verbally on one or more occasions in the last one year; all attributed to gender or identity discrimination. Much more than the 37% physical abuse reported in a study conducted by Barboza et al [14]. Similarly, the prevalence of gender based institutional discrimination and bigotry especially in the employment for this cornered community was around 90%, much more than 39% to 47% reported in other studies [8,9,14]. This shows that developed countries have started accepting their identity. Age at first forced sex was around 15 years which was comparable with the study by Saleem et al [17]. where average age was 14 years. The literature review showed an association between multiple forms of victimization and suicide [14,18,19]. Research especially including transgender community uncovers associations between experiencing interpersonal victimization and sexual discrimination and both suicidal and non-suicidal self-injury [20]. In this current study, we investigated the independent effects of institutional discrimination, discriminatory victimization and non-discriminatory victimization on attempting suicide versus thinking about it or doing neither; selling sex and using substance abuse. Our results support the connection between victimization and suicidal ideation; highlighting physical/verbal abuse due to gender identity increases suicide risk; consistent with previous research by Baiocco et al [21] and Russel et al [22].

In addition, multivariate model after controlling for a number of sociodemographic and psychosocial characteristics shows; participants of our study who experience physical victimization due to their gender identity are more likely to engage in suicidal behavior compared to the individuals who are either not physically victimized or who are physically victimized but do not attribute that experience to gender. This is consistent with findings of previous research in America [14] and Italy [21]. However, non-discrimination-based physical abuse did not show any association with suicide risk, which was not the case in other studies [14,18]. It was evident from our findings that the alleged /so called social and religious norms of our country force exclusion of transgender from performing a role of normalcy [1,13]. Unfortunately, this social exclusion starts from their early childhood till old age [15,17]. In this current study unemployment was much higher (94.4%) as compared to 44% seen in USA [14]. Understanding the results of this social exclusion, it is not hard to appreciate that because of lack of educational, occupational and social opportunities; they are left with no other means of earning a living but to opt for ways not acceptable religiously or in the society i.e. begging, dancing and even selling sex [1,15] Field et al [18] and Baiocco et al [21] also put forth the indicators of social exclusion leading to unfair means of earning. Unfortunately, in countries like Pakistan with majority living in low socio economic conditions; children exhibiting sexual confusions eventually end up with the hijra community. [15] These closed communes act as individual family units; with the Guru serving the role of decision maker while hijras work as income generators [1,12]. In the resent past due to the rapid increase in inflation & their limited role in ceremonies; these income generators find themselves in constant struggle for economic sustainability [23]. Fear of being ridiculed and sexually harassed don't let them go out and work like other men of the community [1,12]. On top of that, lack of educational and occupational opportunities, make it even harder for them to make both ends meet. Hence for the struggle of sustenance this cohort has no other option but to resort to commercial sex work, findings consistent with our study [1,15].

Personality development of any individual needs family support. Lack of contact and support with their original families lead to many psychological and social problems [10]. It has been observed in many studies that this lack of support especially from families, relatives and community members can be one of the reasons why the transgenders are propelled into the vicious cycle of social exclusion; leading them to high risk behaviors like commercial sex work and drug abuse, begging and even suicidal tendency [1,12,15]. Lack of support and discrimination by family was also observed in our study. Moreover, no health education sessions or psychotherapy sessions were reported by our cohort. Housing instability and discrimination at the hospitals was also reported in our study. Wounded of gender identity; people charge them for homosexuality or sex business which is usually regarded as a taboo, with most of it taking place in secrecy [15]. Transgender carrying this impression as providers of “Cheap Sex” and prostitutes; landlords avoid giving them house on rent [1,12,16]. Similarly, transgender being scared that doctors would never take them seriously and would mock them as everyone else does; fail to visit a health care professional. However, who visited a doctor were mostly satisfied (62%). Majority respondents therefore choose alternative medicine for treatment of any ailments, which was highlighted in other study done in Rawalpindi [1,17] and Lahore [10]. Attempted suicide in our sample was only 18% much less than the suicide attempt percentages of studies conducted by Khan et al [12], Barboza et al [14] and House et al [20]; even than the reasons should be probed. However, this 18 % stats are much higher than our national figures [24,25]. Low suicide attempt rate may be because of our religion, which does not allow suicide. Studies conducted by Barboza et al [14], Seedat et al [19] and Baiocco et al [21]. reported forced sex and substance abuse to be significantly related to suicide attempts which was not seen in our study. Discussing a sensitive topic may have few limitations. Participants might not have given exact information regarding selling sex because of question's sensitive nature. Abuse by Gurus would not have been freely reported as interviews were taken in their premises. Use of a cross-sectional study design does not allow us to establish temporality and we cannot make conclusions about causality. Lastly, we did not calculate depression scores which is important predictor for suicidal risk. Nevertheless, given the scarcity of data on a population who has had to struggle for acceptance and security, we believe the strengths of this study outweigh its limitations.

## Conclusion

Our study concludes that social exclusion is one of the many factors that are forcing the transgender community to indulge into inappropriate conducts/habits. Promoting accepting environments and decreasing gender-based prejudice is necessary to improve the adjustment of sexual minorities. Accommodating this group into the mainstream so that the element of social exclusion can be eliminated is very important. Our findings strongly suggest that interventions aimed at increasing social inclusion, reducing gender based discrimination, violence and physical abuse and facilitating access to quality medical care should be considered as part of a comprehensive approach for preventing risky behavior in trans populations.

### What is known about this topic

- Transgender community is socially not accepted in Pakistan;
- Selling sex is common.

### What this study adds

- Reasons why they are socially excluded and their effects on transgenders;
- Suicidal tendency is increasing in this group because of our exclusion/attitude.

## Competing interests

The authors declare no competing interest.
